# BMP12 induces tenogenic differentiation of adipose-derived stromal cells

**DOI:** 10.1371/journal.pone.0077613

**Published:** 2013-10-14

**Authors:** Hua Shen, Richard H. Gelberman, Matthew J. Silva, Shelly E. Sakiyama-Elbert, Stavros Thomopoulos

**Affiliations:** 1 Department of Orthopaedic Surgery, Washington University, St. Louis, Missouri, United States of America; 2 Department of Biomedical Engineering, Washington University, St. Louis, Missouri, United States of America; University of Rochester, United States of America

## Abstract

Adipose-derived stromal cells (ASCs) are pluripotent cells that have the capacity to differentiate into tendon fibroblasts (TFs). They are abundant in adults, easy to access, and are therefore an ideal cell source for tendon tissue engineering. Despite this potential, the molecular cues necessary for tenogenic differentiation of ASCs are unknown. Unlike other bone morphogenetic proteins (BMPs), BMP12, BMP13, and BMP14 have been reported to be less osteo-chondrogenic and to induce tendon rather than bone formation *in vivo*. This study investigated the effects of BMP12 and BMP14 on ASC differentiation *in vitro*. In canine ASCs, BMP12 effectively increased the expression of the tendon markers scleraxis and tenomodulin at both mRNA and protein levels. Consistent with these results, BMP12 induced scleraxis promoter driven-GFP and tenomodulin protein expression in mouse ASCs. Although BMP12 also enhanced the expression of the cartilage matrix gene aggrecan in ASCs, the resulting levels remained considerably lower than those detected in tendon fibroblasts. In addition, BMP12 reduced expression of the bone marker osteocalcin, but not the osteogenic transcription factor runx-2. BMP14 exhibited similar, but marginally less potent and selective effects, compared to BMP12. BMPs are known to signal through the canonical Smad pathway and the non-canonical mitogen-activated protein kinase (MAPK) pathway. BMP12 triggered robust phosphorylation of Smad1/5/8 but not Smad2/3 or p38 MAPK in ASCs. The effect was likely conveyed by type I receptors ALK2/3/6, as phosphorylation of Smad1/5/8 was blocked by the ALK2/3/6 inhibitor LDN-193189 but not by the ALK4/5/7 inhibitor SB-505124. Moreover, ALK6 was found to be the most abundant type I receptor in ASCs, with mRNA expression 100 to 10,000 times that of any other type I receptor. Collectively, results support the conclusion that BMP12 induces tenogenic differentiation of ASCs via the Smad1/5/8 pathway.

## Introduction

Tendon injuries are a common cause of pain and disability. Over 130,000 patients undergo operative tendon repair per year in the United States [1], resulting in significant health care costs and social burden. Despite advances in the treatments of tendon injuries, the clinical outcomes are often poor. Repaired tendons are materially and functionally inferior to healthy tendons, even in the long term, and recurrence of injury is frequent [2,3]. The poor clinical outcomes are largely due to the inherent limitations of tendon tissues, which are relatively hypocellular and hypovascular and thus fail to regenerate. Therefore, cell-based therapies are an attractive future direction for clinical repair of tendon injuries. 

Tendon fibroblasts (TFs) build and maintain the extracellular matrix of tendons and play an important role in tendon repair. Although these fully differentiated cells are ideal candidates for cell-based therapy of tendon injuries, preparation of TFs requires operative removal of healthy tendon tissue followed by isolation and expansion of TFs in culture. The surgery may cause donor site morbidity and expansion of TFs in culture may take an unacceptably long time due to the low initial yield and limited expansion potential of primary TFs [2,3]. These concerns limit the potential of TFs in tendon repair. Bone marrow-derived mesenchymal stromal cells (BM-MSCs) are capable of self-renewal and multi-lineage differentiation in response to specific molecular and physical cues [4,5], and are therefore promising cell source for tendon repair. Nevertheless, collecting bone marrow is a painful procedure. Due to the limited availability of bone marrow and the low frequency of MSCs in bone marrow cells, it may take significant time to expand BM-MSCs in culture to obtain a sufficient number of cells. Moreover, extensive culture expansion may lead to genomic alterations that affect cell function [5,6]. Adipose-derived stromal cells (ASCs) were recently found to contain a subpopulation of multipotent stem cells [7]. In contrast to bone marrow, adipose tissue is typically abundant in adults. Surgical collection of adipose tissue causes little discomfort and donor site morbidity. Furthermore, ASCs proliferate well in culture and are minimally immunogenic [8]. Together, these features make ASCs excellent candidates for cell-based tendon repair.

The molecular cues that induce tenogenic differentiation of ASCs have yet to be identified. Bone morphogenetic protein (BMP) 12, 13, and 14, also known as growth differentiation factor (GDF) 7, 6, and 5 and cartilage-derived morphogenetic protein (CDMP) 3, 2, and 1, are three closely related members of the transforming growth factor beta (TGFbeta) superfamily [9–11]. Unlike other BMPs, they possess low capacity for inducing chondro-osteogenesis [11–15] and have been reported to induce ectopic formation of tendon/ligament-like tissue *in vivo* [11,16]. These growth factors therefore hold promise for use as tenogenic cues for ASCs. 

To evaluate the tenogenic potential of BMP12, ASCs were isolated from canine and mouse subcuticular fat tissues. The dose and time effects of BMP12 on ASC differentiation were examined using a panel of known tendon markers. Selected experiments were also performed using BMP14 for comparison to the literature. Furthermore, the BMP12-induced signaling pathway was explored in ASCs. Results from this study advance current understanding of tenogenic signals for ASCs and provide a basis for future cellular and molecular approaches for tendon tissue engineering and repair.

## Materials and Methods

### Animals and reagents

All experimental procedures were conducted in accordance with the guidelines established by the National Academy of Sciences and overseen by the Animal Studies Committee at Washington University in St. Louis. Female adult mongrel dogs, weighing 20 to 30 kg, were purchased from Covance (Denver, PA). ScxGFP transgenic tendon reporter mice [17], kindly provided by Dr. Ronen Schweitzer at Oregon Health & Science University, were bred in the Washington University animal facility. Recombinant human BMP12 and BMP2 were acquired from Pfizer (New York, NY). All other reagents were purchased from Sigma-Aldrich (St. Louis, MO) unless specified elsewhere.

### Study design

ASCs from a large animal model (canine) were used due to the translational potential for clinical practice [18–21]. A total of seven dogs that gave rise to seven ASC isolations were used in this study. Two ASC isolations were used for the colony forming unit-fibroblast (CFU-F) assay and for surface marker determination (n = 2 for each experiment). Two additional isolations were used to evaluate the differentiation potential of ASCs (adipogenic, osteogenic, and chondrogenic differentiation; n = 2 for each). The remaining three ASC isolations were used in three independent experiments to study the dose and time effects of BMP12 and BMP14 on ASC differentiation. ASCs from each isolation were treated in duplicate. One set of treated cells was used for RNA isolation followed by quantitative real-time RT-PCR for tendon, cartilage, and bone marker gene expression (n = 3); the other set of treated cells was used for protein isolation and the subsequent Western blot analysis of tendon markers (n = 3). In addition to ASCs, TFs from the corresponding animals were also isolated and cultured in parallel as positive controls. 

ScxGFP transgenic mice were used to further corroborate findings from the canine model. In these mice, the expression of green fluorescent protein (GFP) is driven by the tendon-specific scleraxis promoter, thus indicating the activation of tenogenic signaling [17]. ASCs were isolated from nine ScxGFP mice at the age of 8 weeks. Two of the ASC isolations were used for the CFU-F assay. Three isolations were treated with BMP12 and analyzed by immunofluorescent staining for expression of GFP and tenomodulin and by Western blot for phosphorylation of Smad proteins and p38 in the presence or absence of activin-like kinase (ALK) inhibitors (n = 3 for each analysis). The remaining four ASCs isolations were not treated and used to determine relative quantities of ALKs and type II BMP receptor (BMPRII) mRNA in undifferentiated ASCs by real-time RT-PCR (n = 4). Non-treated TFs were used as positive control to compare GFP expression in corresponding ASCs.

### Cell isolation and culture

Canine and mouse ASCs were isolated from subcutaneous fat. The fat tissues (5 g from canine and 1 g from mouse) were minced into a fine slurry, digested with 0.2% collagenase A (Roche Diagnostics, GmbH, Mannheim, Germany) in PBS at 37°C for up to 2 h, and then centrifuged at 250 x g for 10 min. The pellets were re-suspended in minimum essential medium alpha (alpha-MEM; Meditech Inc, Manassas, VA) and filtered through a 70 µm nylon mesh to remove undigested tissues. The filtrates, containing stromal vascular fraction (SVF) cells, were re-centrifuged as above. The SVF cells were then re-suspended in alpha culture medium containing 10% FBS, 100 unit/ml penicillin, 100 µg/ml streptomycin, and 2.5 µg/ml amphotericin B in alpha-MEM and subsequently cultured in either a T150 (for canine cells) or a T75 (for mouse cells) flask. Within the next 24 to 48 h, ASCs were selected by removing unattached cells with two PBS washes. The plastic adherent ASCs were then maintained in alpha culture medium and passaged when they were 80-90% confluent at a density of 6000 cells/cm^2^. 

Canine and mouse TFs were prepared from flexor digitorum profundus and tail tendons, respectively. Canine tendon tissues were diced into 2-3 mm cubes, while murine tail tendon fascicles were cut into a few fragments. TFs of either species were then isolated from respective tissues and cultured as described for ASCs. 

All of the cells were used for experiments at passage 2 unless specified elsewhere.

### CFU-F assay

The CFU-F assay was performed according to the method described by Schellenberg et al [22]. Briefly, SVF cells isolated from fat tissue were plated in 96-well plates at densities of 3000, 1000, 300, 100, and 30 cells per well (48 replicas per density) and cultured in alpha culture medium for 2 weeks. The cultures were then stained with 0.1% crystal violet solution. Colony formation was defined by crystal violet staining that covered at least 50% of surface area of a 96-well. CFU-F frequency was determined with L-Calc Software (STEMCELL Technologies) based on Poisson statistics.

### Flow cytometry

To quantify surface marker expression on canine ASCs, cells were dislodged by trypsin-EDTA and stained with FITC-conjugated anti-CD44 (eBioscience, clone YIX337.8) and APC-conjugated anti-CD90 (eBioscience, clone YKIX337.2) antibodies in PBS containing 1% FBS for 30 min at room temperature. After washes, the stained cells were immediately examined with FACSCanto II (BD Biosciences) using FACSDiva software. The cells were gated based on their forward and side scatter properties to exclude debris and doublets. Data analysis was performed with FlowJo software (Tree Star, Inc.) using unstained cells as a negative control to compare CD44 and CD90 positive populations.

### Induction of ASC differentiation

To induce tenogenic differentiation, ASCs were plated in differentiation medium (alpha-MEM supplemented with 2% FBS) at a density of 1 × 10^4^ cells/cm^2^. On the following day, the cells were treated with the indicated doses of either BMP12 or BMP14 (R&D Systems, Minneapolis, MN) in the same medium for up to 14 days with medium change every 2 to 3 days. 

Adipogenic differentiation was induced in 100% confluent ASCs by alternatively growing the cells in adipogenic induction medium (Lonza, Walkersville, MD) and adipogenic maintenance medium (Lonza) for two cycles. In each cycle, the cells were first grown in adipogenic induction medium for 7 days with medium change every 2 to 3 days, followed by 3 days of culture in adipogenic maintenance medium. Afterward, the cells were washed with PBS, fixed in 10% neutral buffered formalin, and stained with Oil Red O for lipid droplets. 

Chondrogenic differentiation was conducted by growing ASC pellets (2.5 × 10^5^ cells per pellet) in chondrogenic induction medium (Lonza) containing 10 ng/ml TGFbeta3 (R&D Systems) with medium change every 2 to 3 days for up to 28 days. The pellets were then fixed as above, embedded in paraffin, sectioned to 5 µm thick, and stained with Alcian Blue. 

For osteogenic differentiation, ASCs were grown in 6-well plate pre-coated with collagen I (BD Biosciences, Bedford, MA) in osteogenic induction medium (alpha-MEM supplemented with 10% FBS, 50 mg/L ascorbic acid, 10 mM beta-glycerophosphate, 100 unit/ml penicillin, and 100 µg/ml streptomycin) at a density of 3 × 10^3^ cells/cm^2^ for 28 days with medium change every 2 to 3 days. The culture was then fixed and stained with 2% Alizarin Red.

### RNA isolation and real-time RT-PCR

Cultured ASCs and TFs were directly lysed in culture plates with Trizol Reagent (Life Technologies, Carlsbad, CA) according to the manufacturer’s instructions. Chloroform was then added to the cell lysates (0.2 ml per ml of Trizol). After vigorous shaking and a brief incubation (2-3 min) on a rotator, the samples were added to Phase Lock Gel (5 Prime GmbH, Hamburg, Germany) and centrifuged at 13,000 × g for 6 min at 4 °C. RNA in the resulting upper aqueous phase was purified with RNeasy Mini Spin Column (Qiagen Sciences, MD, USA) according to the manufacturer’s instructions. Potential genomic DNA contamination was eliminated by treating RNA samples with DNase I (Qiagen) during purification. 

Real-time RT-PCR was performed in two steps. In the first step, total RNA (500 ng) was reversely transcribed into first-strand cDNA in a 20 µl reaction with random primers using the SuperScript VILO cDNA Synthesis Kit (Life Technologies) according to the manufacturer’s instructions. The resulting cDNA reaction was diluted with 30 µl of nuclease free water. In the second step, SYBR Green-based real-time PCR was performed. The PCR, containing 1 µl of the diluted cDNA reaction from the first step, 1 × QuantiTect Primers (Qiagen) or 1 µM customized forward and reverse primers (Table 1), and 1 × SYBR Green PCR Master Mix (Applied Biosystems, Warrington, UK) in 20 µl total volume, was carried out in triplicate with StepOnePlus Real-Time PCR System (Applied Biosystems). The PCR program was set as the follows: 10 min at 95 °C for 1 cycle; 15 sec at 95 °C and 40 sec at 60 °C for 40 cycles. A melt curve analysis was performed at the end of each SYBR Green PCR. The efficiencies of all the primers were over 90%. The relative quantity of target gene expression was analyzed using the comparative C_T_ (2^-∆∆C^_T_) method. GAPDH was used as endogenous reference gene. The PCR efficiencies between target genes and GAPDH were approximately equal. The relative abundance of CD14 and CD34 mRNAs were expressed as fold change related to mRNA levels of ASCs at passage 0. In the ASC differentiation study, the results are shown as fold change related to control ASCs that received vehicles only. For the expression of ALKs and BMPRII in non-treated ASCs, the data were compared to a mouse reference RNA sample purchased from Agilent Technologies. 

**Table 1:** Customized primers for real-time PCR ^**a**^.

**Canine**	**Forward primer**	**Reverse primer**	**Reference/Accession #**
*SCX*	5'-AAGCTCTCCAAGATCGAGACACTG-3'	5'-AAGAAGGGCCCAGAGTGGCA-3'	XM_003431821
**Mouse**	**Forward primer**	**Reverse primer**	
*ALK1*	5’-GGCCTTTTGATGCTGTCG-3’	5'- ATGACCCCTGGCAGAATG-3'	Luo et al, 2010
*ALK4*	5'-CCCCCTTGTTGTCCTCCT-3'	5'-GGCCCCATCTGTCTCACA-3'	Luo et al, 2010
*ALK5*	5'-TGTGCACCATCTTCAAAAACA-3'	5'-ACCAAGGCCAGCTGACTG-3'	Luo et al, 2010
*ALK7*	5'-ATGGCTCCCGAAATGCTTGA-3'	5'-ACAACTCCTCCAACTGAACACC-3'	NM_001111030

^a^ Unlisted real-time PCR primers were purchased from Qiagen.

### Western blot assay

To examine the expression of tenogenic markers, ASCs were cultured and treated with BMP12 as described above. To study Smad phosphorylation, ASCs were cultured in 12-well plates at a density of 1 × 10^5^ cells per well and starved in differentiation medium overnight. On the following day, the cells were treated with either BMP12 (1000 ng/ml), BMP2 (200 ng/ml), or TGFbeta3 (10 ng/ml) for the indicated periods in duplicates. The dose of BMP12 was chosen based on the results from this study, which resulted in the strongest tenogenic effects on ASCs among all the doses examined. The doses for BMP2 and TGFbeta3 were selected based on those reported in literatures [23–25]. In cases when the inhibitor LDN-193189 (Stemgent, San Diego, CA) or SB-505124 was applied, the cells were pretreated with either of the drugs for 15 min prior to the addition of growth factors.

At the end of treatments, the cells were washed twice with PBS and then lysed in ice-cold RIPA buffer containing 1 mM DTT and protease inhibitor cocktail (Roche Diagnostics, Indianapolis, IN). The resulting whole cell lysates were pooled from the duplicated wells and concentrated with Amicon Ultra-0.5 Centrifugal 10 K Filter (Millipore Ireland Ltd, Cork, Ireland). The protein concentrations of these concentrated samples were determined with a Pierce BCA Protein Assay Kit (Thermo Scientific, Rockford, IL) according to the manufacturer’s instructions. 50 µg of each sample were then separated on 4% to 12% NuPAGE Bis-Tris Mini Gel (Life technologies) under reducing conditions and subsequently transferred to nitrocellulose membranes (0.2 µm; Thermo Scientific). The membranes were blocked with 5% BSA in TBST buffer containing 10 mM Tris-HCl (pH 8.0), 150 mM NaCl, and 0.1% Tween 20 and incubated with one of the following rabbit primary antibodies: phospho-Smad1/5/8 (Cell Signaling, Danvers, MA), phospho-Smad2 (Cell Signaling), phosphor-Smad3 (Cell Signaling), SCXA (Abcam, Cambridge, MA), or Tenomodulin (Santa Cruz Biotechnology) at 4°C overnight. After three washes with TBST, the membranes were incubated with Peroxidase-conjugated Anti-Rabbit IgG (H+L) (Jackson ImmunoResearch, West Grove, PA) for 1 h, and then washed and detected in Pierce ECL Western Blotting Substrates (Thermo Scientific). After exposure to X-ray film, the membranes were stripped, re-blocked, and re-probed with either of the following rabbit antibodies specific for phospho-p38 (Cell Signaling), beta-Actin (Abcam), or total Smad1 (Cell Signaling), as described above. Semi-quantification of band volume was performed with Quantity One 4.6.5 software (Bio-Rad) after subtraction of background and normalized by beta-Actin sample loading control. 

### Immunofluorescence staining

For tenogenic protein expression, cultured ASCs derived from ScxGFP tendon reporter mice were fixed with 4% paraformaldehyde in PBS. After three washes with PBS, the cells were permeabilized with 0.5% Triton X-100 in PBS, blocked with 5% normal donkey serum (Jackson ImmunoResearch) in PBST (PBS containing 0.1% Triton X-100), and incubated with rabbit anti-Tenomodulin antibodies (Santa Cruz) at 4°C overnight. After three washes with PBST, the cells were incubated with Cy3-conjugated Donkey Anti-Rabbit IgG (H+L) (Jackson ImmunoResearch) for 1 h at room temperature and then counterstained with bisBenzimide H33258. 

For surface marker expression, canine and mouse ASCs were fixed as above, blocked with 5% normal donkey serum in PBS, and then either directly stained with FITC-conjugated rat anti-CD44 antibodies (eBiosciences, clone IM7), or indirectly stained with mouse anti-CD29 (BD Biosciences, clone 18/CD29) or mouse anti-CD90 antibodies (BD Biosciences, clone 5E10) at 4°C overnight followed by incubation with Dylight 488-conjugated Donkey Anti-Mouse secondary antibodies (Jackson ImmunoResearch) for 1h at room temperature. After thorough wash with PBS, the cells were counterstained with bisBenzimide H33258.

### Statistics

Statistical analysis was performed by ANOVA and Fisher’s PLSD test using StatView 5.0 (SAS Institute Inc.). Statistical significance was set at P < 0.05. All data are shown as mean ± standard deviation unless otherwise noted. 

## Results

### Characterization of ASCs

Mesenchymal stromal cell frequencies can be described using the CFU-F assay [26]. The CFU-F frequency of canine and mouse SVF cells directly isolated from fat tissues was determined in 96-well using a limit dilution method [22]. The assay revealed an average CFU-F frequency of 1 in 90 for canine cells (Table 2), which was more than three times of that detected in mouse cells (1 in 339) and at least 100 times of that previously reported on bone marrow mononuclear cells [27,28].

**Table 2:** CFU-F frequency of canine and mouse ASCs.

**ASC donor**	**Gender**	**Frequency**	**Frequency + 1 S.E.**	**Frequency - 1 S.E.**
dog-1	F	1 in 86	1 in 76	1 in 97
dog-2	M	1 in 94	1 in 107	1 in 122
mouse-1	M	1 in 339	1 in 300	1 in 383
mouse-2	M	1 in 338	1 in 296	1 in 387

**Figure 1:**
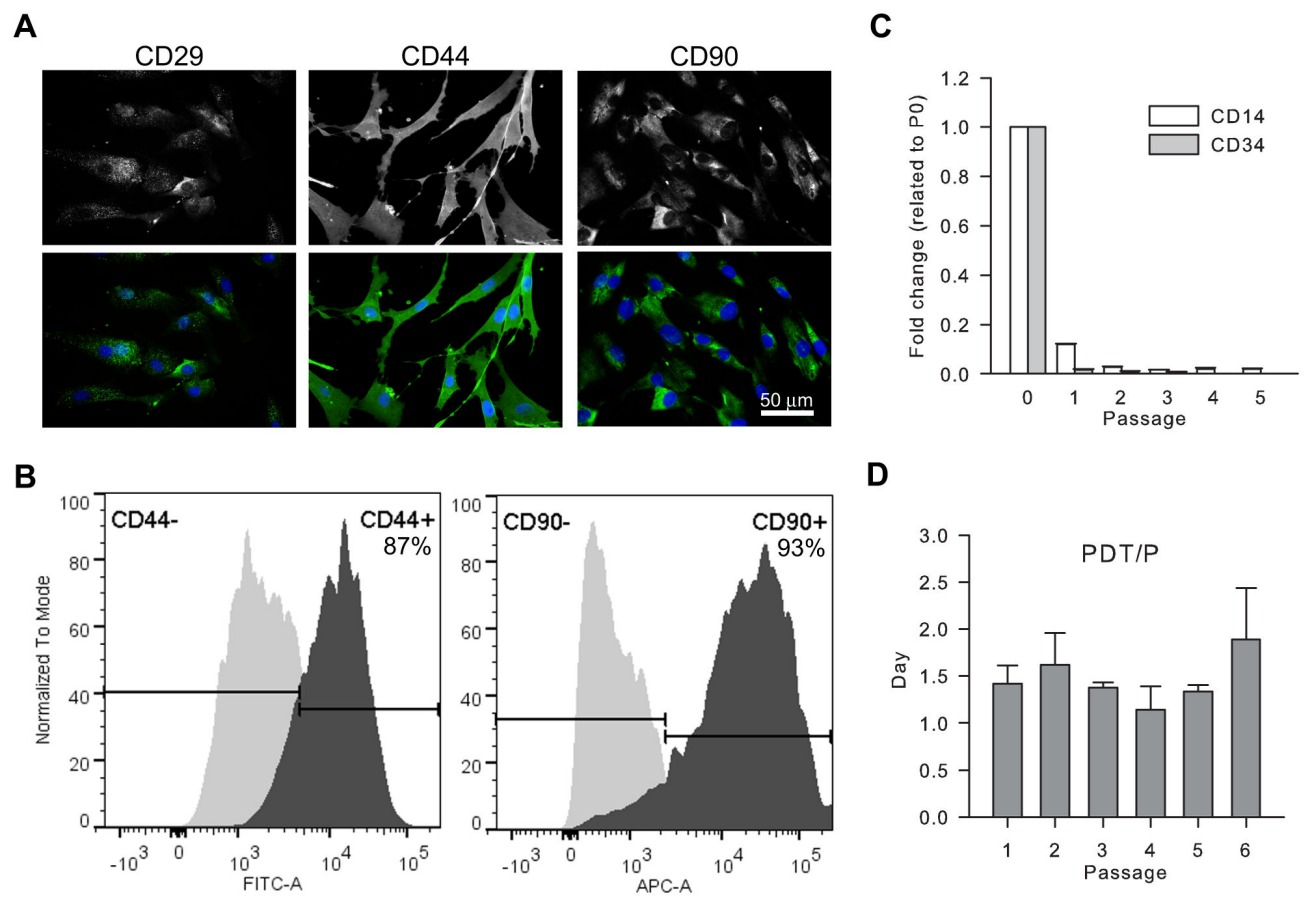
Characterization of canine ASCs. Representative images show positive immunofluorescent staining of stromal markers CD29, CD44, and CD 90 on almost all passage 2 ASCs (A). Representative histograms from flow cytometry analysis reveal dominant expression of CD 44 and CD90 on passage 6 ASCs (B). The mRNA expression of hematopoietic markers CD14 and CD34 in ASCs was dramatically reduced with progressive passages (C). The population doubling time (PDT) of canine ASCs was is shown through passage 6 (D).

The surface marker expression on canine ASCs was evaluated by immunofluorescent staining. Consistent with previous reports [29–31], mesenchymal stromal cell markers CD29, CD44, and CD90 [32] were detected on nearly all canine (Figure 1A) and mouse (data not shown) ASCs at passage 2, with distinct expression patterns. Prevalent expression of CD44 (87.0% ± 0.28%) and CD90 (88.1% ± 7.35%) were also detected on passage 6 canine ASCs by flow cytometry analysis (Figure 1B). 

Low levels of hematopoietic markers such as CD34 have been reported in human ASCs [30]. To overcome the limitation of rare-event analysis by flow cytometry, we used the more sensitive quantitative real-time PCR [33] to evaluate the expression of CD14 and CD34 in canine ASCs from passage 0 (after initial plating) to passage 5. Although both mRNAs were detected by real-time RT-PCR in passage 0 ASCs, their expression levels were substantially decreased in ASCs with successive passages and became barely detectable after passage 2 (Figure 1C). This temporal change is in consistent with that reported in the literature [30].

The differentiation potential of canine ASCs was further assessed using standard differentiation culture conditions. Results confirmed that these cells were able to differentiate into adipocytes, osteoblasts, and chondrocytes, as demonstrated by Oil Red O, Alizarin Red, and Alcian Blue staining under the respective culture conditions (Figure 1). 

The proliferation capacity of canine ASCs was also evaluated from passage 1 to passage 6. The result revealed a stable population doubling time at an average of 1.5 days at each passage (Figure 1D).

Taken together, the data demonstrated that canine ASCs prepared in this study contained colony-forming cells that expressed mesenchymal stromal cell markers and were able to differentiate into multiple cell types. 

### BMP12 and BMP14 induced tenogenic differentiation of ASCs

The dose and time effects of BMP12 and BMP14 on ASC differentiation were investigated using quantitative real-time RT-PCR targeting the tendon markers scleraxis (*SCX*) and tenomodulin (*TNMD*); the tendon matrix genes collagen, type I, alpha 1 (*COL1A1*), collagen, type III, alpha 1 (*COL3A1*), and decorin (*DCN*); the cartilage matrix genes aggrecan (*ACAN*) and collagen, type II, alpha 1 (*COL2A1*); and the bone markers osteocalcin (*BGLAP*, bone gamma-carboxyglutamic acid-containing protein) and runt-related transcription factor 2 (*RUNX2*). In addition to ASCs, gene expression levels in the corresponding non-treated TFs were assessed in parallel as a reference for TF phenotype.

**Figure 2:**
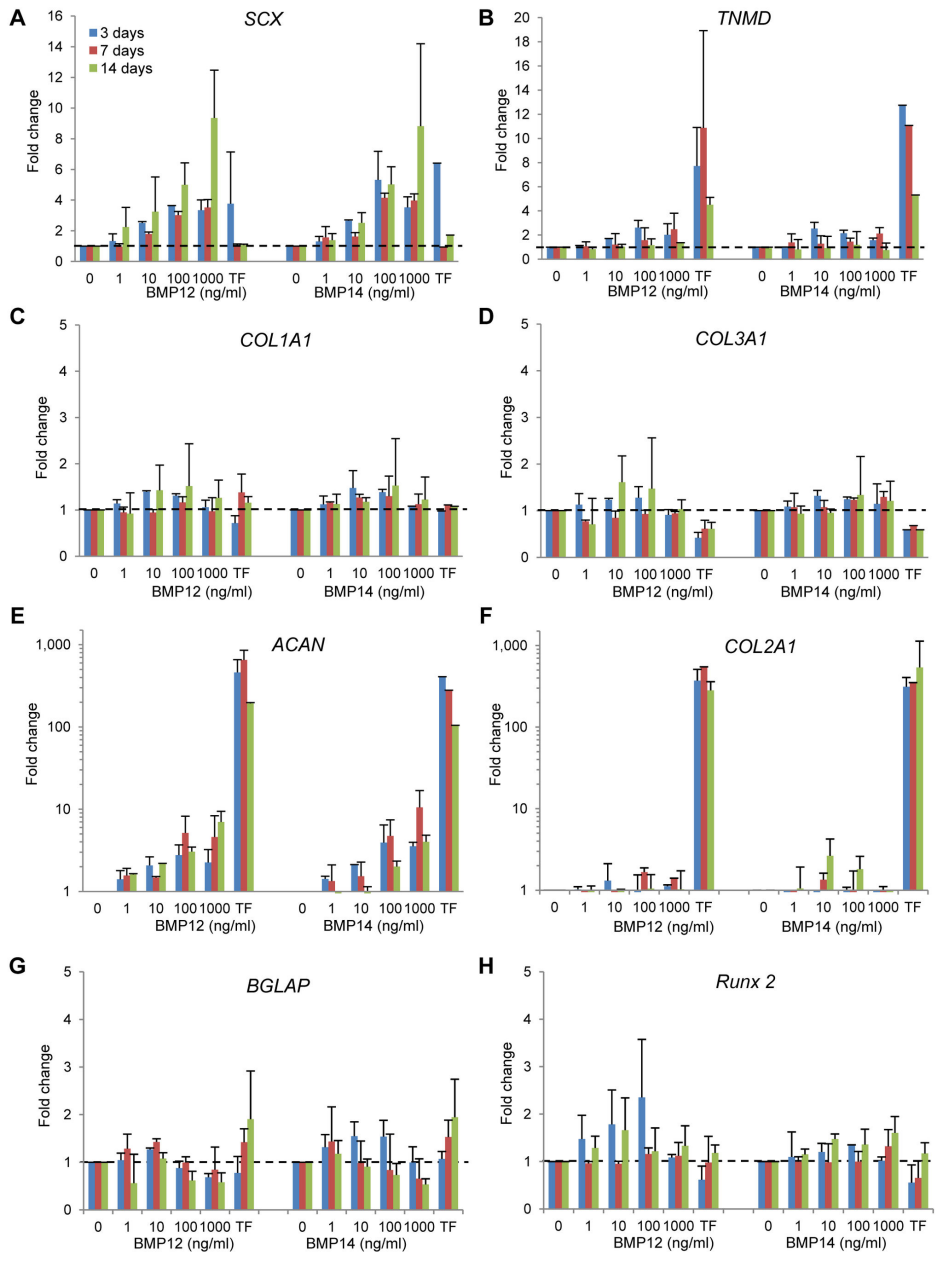
BMP12 and BMP14 induce tenogenic gene expression in canine ASCs. The mRNA expression of the tendon markers *SCX* and *TNMD* was significantly increased in canine ASCs treated with BMP12. In contrast, the expression of the bone marker *BGLAP* was dose- and time-dependently reduced by BMP12. Similar changes in gene expression were detected in BMP14-treated ASCs. The results were determined by quantitative real-time RT-PCR and are shown as fold change related to the expression level of control ASCs (dashed lines). Log scales are used in (E) and (F). The legend in (A) applies to all of the panels.

 SCX is a tendon/ligament-specific transcription factor [34] that positively regulates TNMD expression [35], and TNMD is required for TF proliferation and tendon maturation [36]. Our results revealed that BMP12 dramatically increased *SCX* expression in ASCs by up to 9-fold in a dose- and time-dependent manner (Figure 2A; P < 0.0001 for dose, P = 0.0018 for time). As a result, the *SCX* levels in the BMP12-treated ASCs (100 ng/ml and 1000 ng/ml) were similar to those in TFs. BMP14 also caused a substantial increase in *SCX* expression (Figure 2A). The effect was dose- (P < 0.0001) but not time-dependent (P = 0.18). BMP12 also dose-dependently increased *TNMD* expression in ASCs by up to 3-fold (Figure 2B; P = 0.04). No significant change in *TNMD* was detected in BMP14-treated ASCs (Figure 2B; P = 0.22). Although neither BMP12 nor BMP14 increased *COL1A1* expression in ASCs, the resulting *COL1A1* levels were comparable to those in TFs (Figure 2C). Similarly, BMP12 and BMP14 did not alter *COL3A1* expression in ASCs (Figure 2D). Compared to TFs, *COL3A1* levels in BMP12-treated ASCs were significantly higher at 3 days at 10 and 100 ng/ml doses (P < 0.05) but not at any other time points. For the BMP14-treated ASCs, in addition to 3 days (Figures 2D; 10 and 100 ng/ml; P < 0.05 vs. TFs), higher *COL3A1* levels were also detected at 7 days (Figures 2D; 100 and 1000 ng/ml, P < 0.05 vs. TFs). No changes were found in *DCN* expression after either BMP12 or BMP14 treatment, and the *DCN* levels in the BMP-treated ASCs were similar to those in TFs (Figure 2). 

When examining the expression of cartilage matrix genes, both BMP12 and BMP14 dose-dependently augmented *ACAN* expression (Figure 2E; P < 0.01 for both BMPs). However, the effect was not time-dependent, and the resulting *ACAN* levels in ASCs remained substantially lower than those detected in TFs (P <0.0001 for both BMP12 and BMP14). Neither BMP12 nor BMP14 affected *COL2A1* expression in ASCs (Figure 2F). Similar to *ACAN*, considerably higher levels of *COL2A1* were found in TFs compared to either BMP12- or BMP14-treated ASCs (P < 0.0001). 

With regard to osteogenic gene expression, BMP12 but not BMP14 dose- and time-dependently suppressed the osteoblastic *BGLAP* expression in ASCs by up to 50% (Figure 2G; P = 0.01 for dose, P = 0.02 for time). BMP12 and BMP14 did not affect *RUNX2* expression in ASCs, and no significant difference in *RUNX2* expression between BMP-treated ASCs and TFs were detected (Figure 2H). 

Collectively, the results indicated that both BMP12 and BMP14 were capable of inducing tendon marker gene expression in ASCs. The most effective dose and treatment time for both BMPs were 1000 ng/ml for 14 days, respectively. BMP12 was marginally more potent (based on significant increase in *SCX* and *TNMD*) and selective (based on downregulation of *BGLAP*) than BMP14. Therefore, we focused on BMP12 in the subsequent studies.

**Figure 3:**
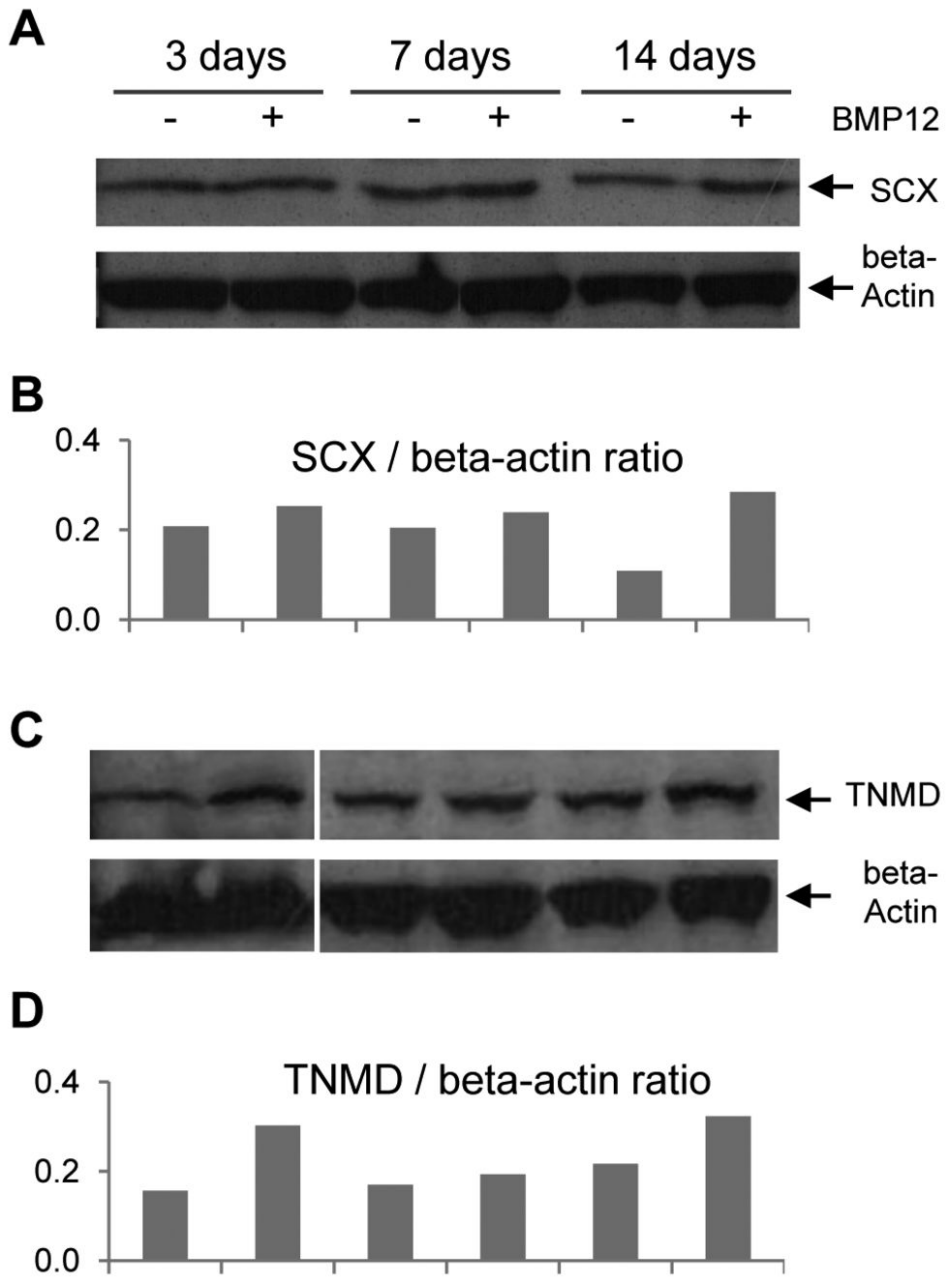
BMP12 increases SCX and TNMD protein expression in ASCs. Representative Western blots (A and C) and semi-quantifications (B and D) of band volumes of the corresponding bands are shown.

Results at the protein level were consistent with the gene expression results. Western blot analysis demonstrated increased SCX expression in BMP12-treated ASCs at all three time points studied, with the highest increase at 14 days (Figures 3A and 3B). Similarly, TNMD expression was increased in ASCs with BMP12 treatment, especially at 3 day and 14 days (Figures 3C and 3D). 

**Figure 4:**
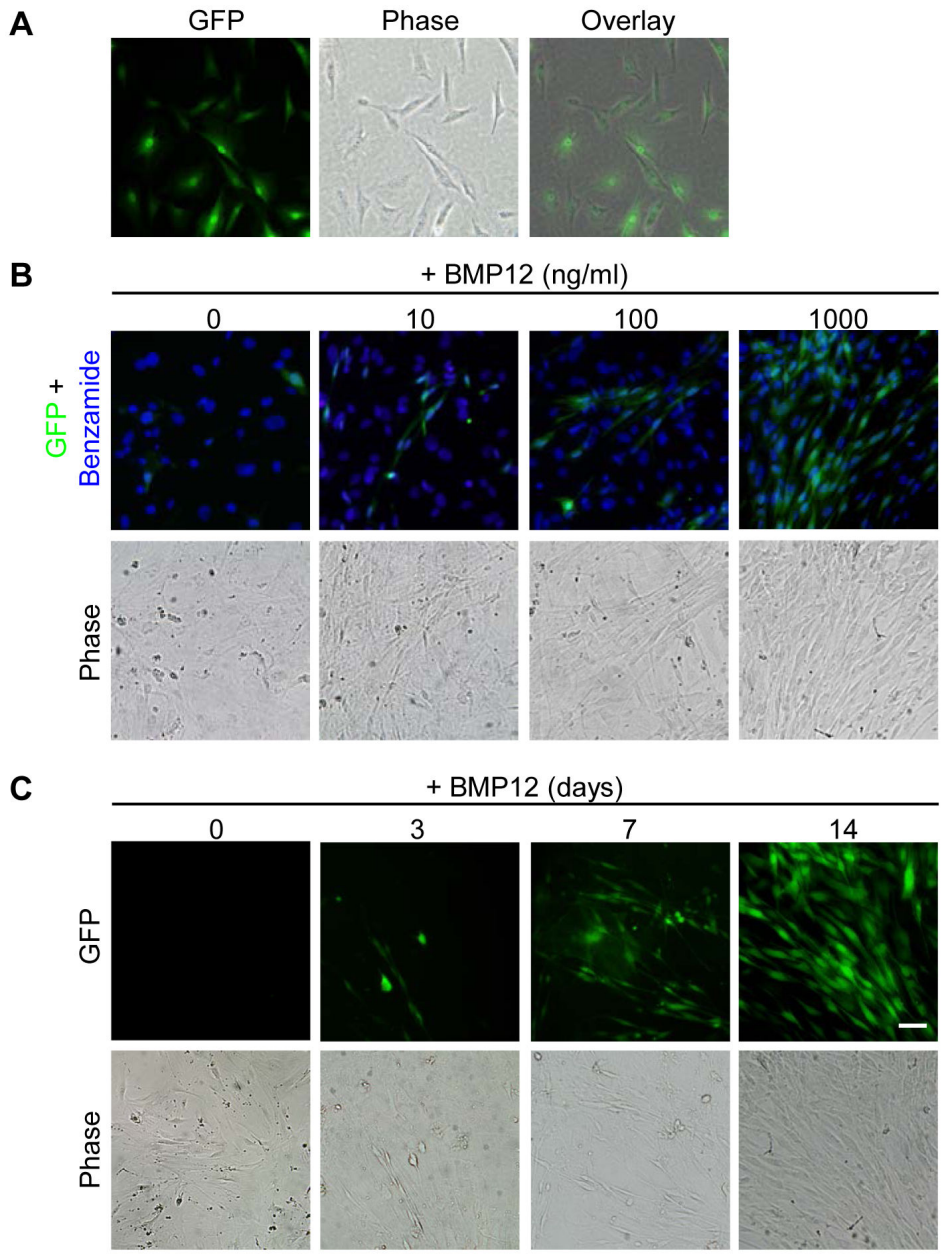
BMP12 induces scleraxis promoter-driven GFP expression in mouse ASCs. GFP was expressed by all the TFs from the ScxGFP transgenic mice in the cell nuclei (A). The number of GFP-positive cells was increased in BMP12-treated ASCs in a dose- (B) and time- (C) dependent manner. Representative fluorescent and phase contrast images are shown. ASCs in (B) and (C) were treated with BMP12 for 14 days and at a concentration of 1000 ng/ml, respectively. The scale bar = 50 µm and applies to all of the panels.

**Figure 5:**
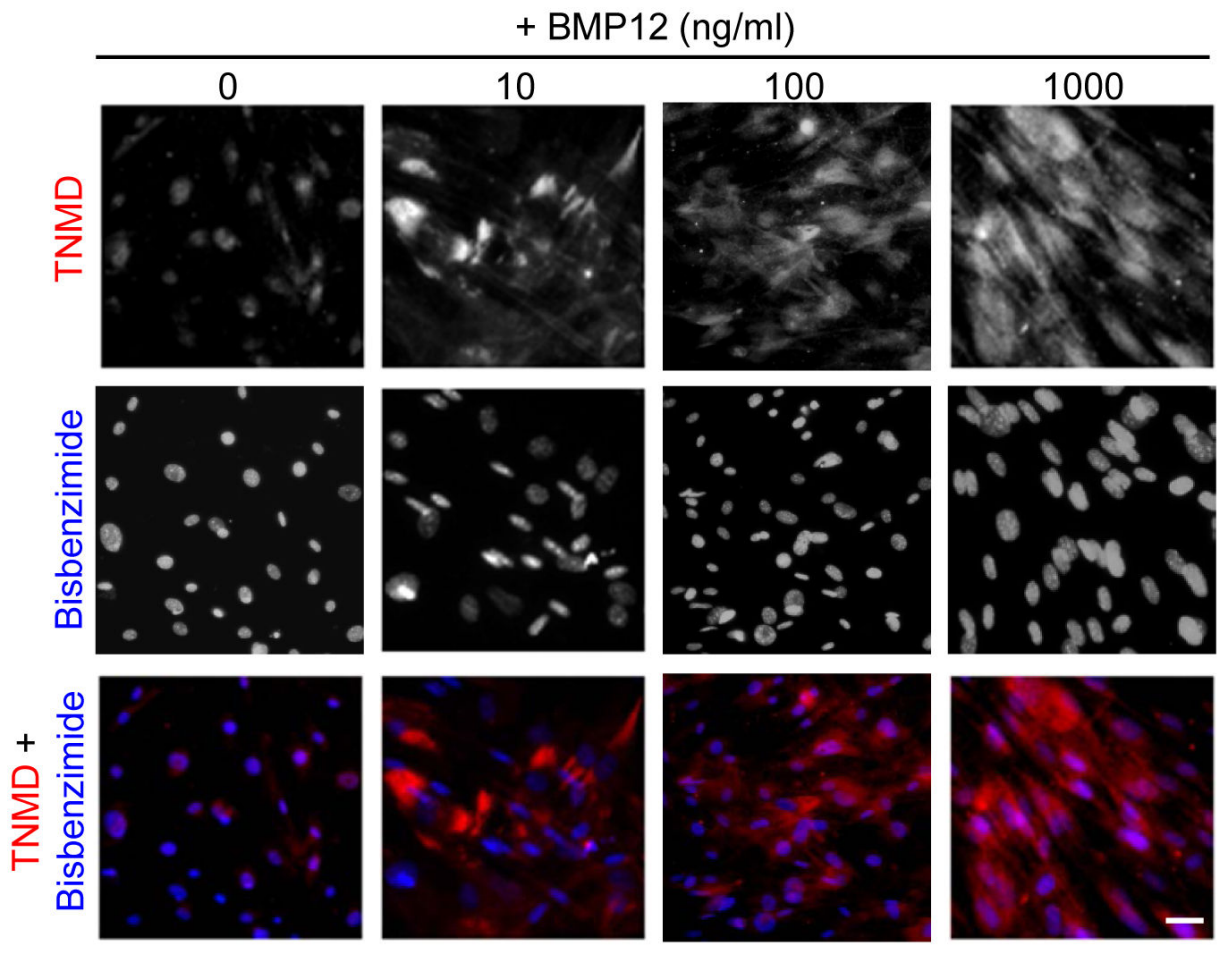
BMP12 increases TNMD protein expression in mouse ASCs. The expression of TNMD protein (in red) in ASCs from ScxGFP transgenic mice was dose-dependently increased after a 14-day treatment with BMP12. Representative fluorescent images are shown. Cell nuclei were counterstained with H33258 (in blue). The scale bar = 50 µm and applies to all panels.

To further validate the findings, the ScxGFP transgenic mice, specifically expressing GFP in tendon tissues under the control of scleraxis promoter [17], were employed in this study. The expression of the ScxGFP tendon reporter in ASCs from these mice was evaluated after BMP12 treatments. As expected, strong GFP expression was present in the cell nuclei of all TFs (Figure 4A), but absent in non-treated undifferentiated ASCs (Figures 4B and 4C, 0 ng/ml). Application of BMP12 induced dose- (Figure 4B) and time-dependent (Figure 4C) increases in GFP-positive cells in ASC culture. The resulting GFP-positive ASCs exhibited spindle-shaped morphology and alignment, forming TF-like cultures by 14 days (Figure 4B). Moreover, immunostaining with TNMD antibodies detected dose-dependent, robust increases in TNMD-immunoreactivity at perinuclear and cytoplasm regions of these BMP12-treated cells (Figure 5). Taken together, our data supported that BMP12 is capable of inducing tenogenic differentiation of ASCs.

### BMP12 induced tenogenic signaling in ASCs via the Smad1/5/8 pathway

**Figure 6:**
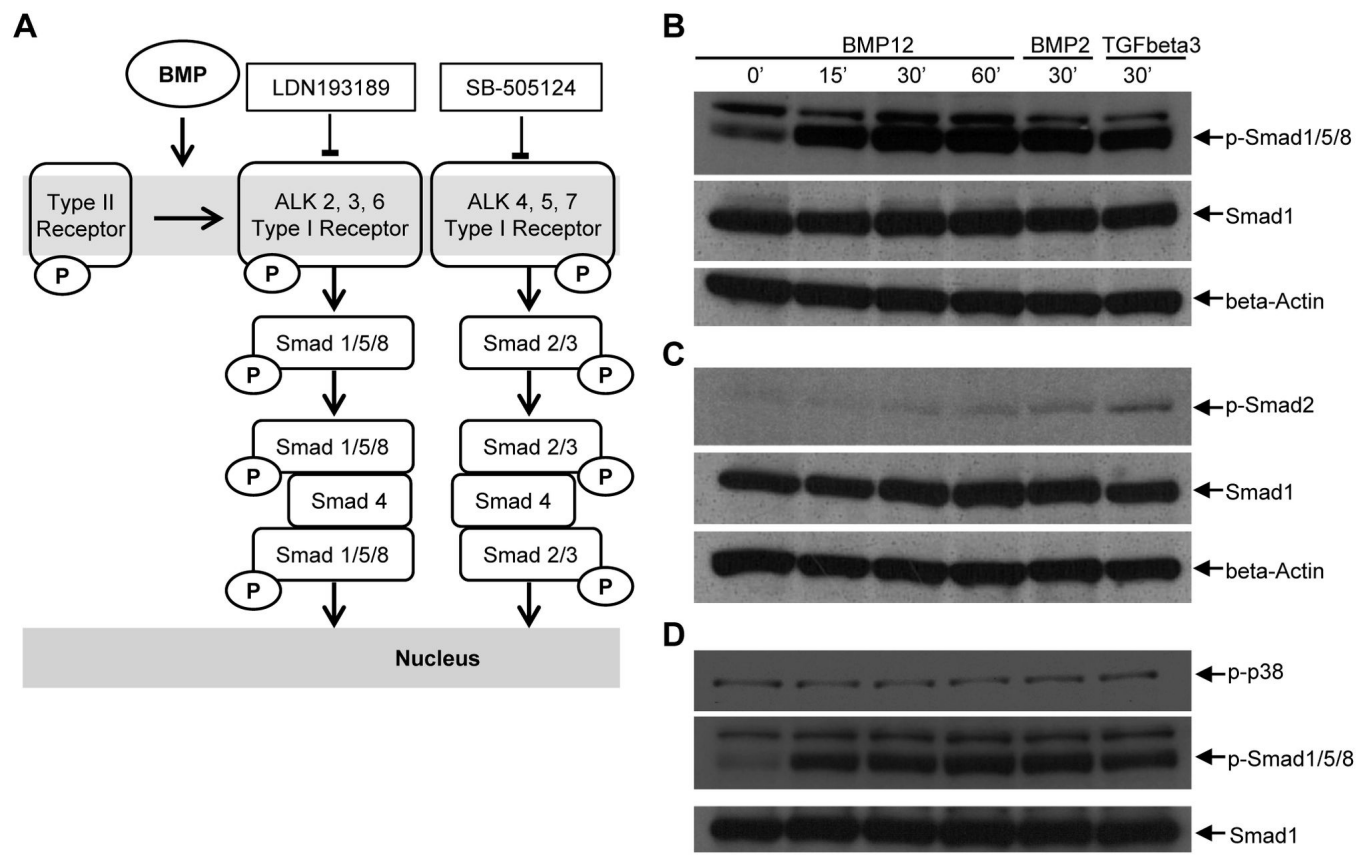
BMP12 activates Smad1/5/8 signaling pathway in ASCs. The interaction of BMPs with type I and type II receptors leads to the transphosphorylation of type I receptors by type II receptors (A). The type I receptors are the signaling receptors that activate Smad signaling pathways. There are seven known type I receptors, namely ALK1 through ALK7. They are typically separated into two groups: ALK1, 2, 3, and 6 phosphorylate Smad1/5/8; ALK4, 5, and 7 phosphorylate Smad2/3. LDN-193189 and SD-505124 are selective inhibitors for ALK2, 3, 6 and ALK4, 5, 7, respectively (A). For simplicity, ALK1 is not shown in the figure. Smad1/5/8 (B) but not Smad2 (C) and p38 (D) in ASCs was phosphorylated by BMP12 (1000 ng/ml) as detected by Western blot. BMP2 (200 ng/ml) and TGFbeta3 (10 ng/ml) were used as positive controls for the induction of phosphorylation of Smad1/5/8 and Smad2, respectively.

Like other ligands in the TGFbeta superfamily, BMPs bind to type I and type II receptors (Figure 6A) [37]. Both types of receptors are transmembrane serine-threonine kinases. Type II receptors are constitutively active, while type I receptors are transphosphorylated and therefore activated by type II receptor upon ligand binding and type I and type II receptor oligomerization. The activated type I receptors subsequently activate the canonical Smad pathways and phosphorylate either Smad1/5/8 or Smad2/3 proteins. The phosphorylated Smad1/5/8 (p-Smad1/5/8) or Smad2/3 (p-Smad2/3) in turn form a heteromeric complex with Smad4 and translocate into nucleus, thus interacting with other transcription factors and regulating target gene expression. 

Although most BMPs activate Smad1/5/8, there are some exceptions, e.g., BMP3 and BMP11 [38]. Furthermore, there is promiscuity of BMPs/TGFbetas with regard to their ligand-receptor relationship [38]. These prior results motivated the question of whether the Smad1/5/8 or the Smad2/3 pathways were activated in ASCs after BMP12 treatment. As shown in Figure 6B, without altering the expression of total Smad1, BMP12 induced robust phosphorylation of Smad1/5/8 within 15 min, and the strong p-Smad1/5/8 signal persisted even after 60 min of treatment. In contrast, BMP12 resulted in little phosphorylation of Smad2 (Figure 6C) and Smad3 (Figure 3), while p-Smad2 and p-Smad3 signals were detected in ASCs treated with TGFbeta3 for 30 min. 

In addition to the canonical Smad pathway, BMPs are known to activate non-Smad pathways such as mitogen-activated protein kinase (MAPK) [39]. Furthermore, the p38 MAPK has been implicated in tenogenesis [40]. To test if p38 was also involved in the BMP12-induced tenogenic differentiation of ASCs, Western blots were performed. There were no detectable increases in phosphorylated p38 (p-p38) in ASCs after BMP12 treatment (Figure 6D). Thus, the Smad1/5/8, but not the Smad2/3 or p38, pathway was activated in ASCs upon BMP12 treatment.

We next asked which type I receptors activate Smad1/5/8 in ASCs. To answer this question, the gene expression profile of all seven type I receptors, namely *ALK1* through *ALK7*, were surveyed together with the type II receptor *BMPRII* in ASCs by quantitative real-time RT-PCR. Interestingly, although all of these receptors were detectable in non-treated ASCs, the expression level of *ALK6* (also known as *BMPR1B*), was 100- to 10,000-fold higher than that of any other receptor evaluated (P < 0.05; Figure 4), and therefore was more likely to interact with BMP12.

**Figure 7:**
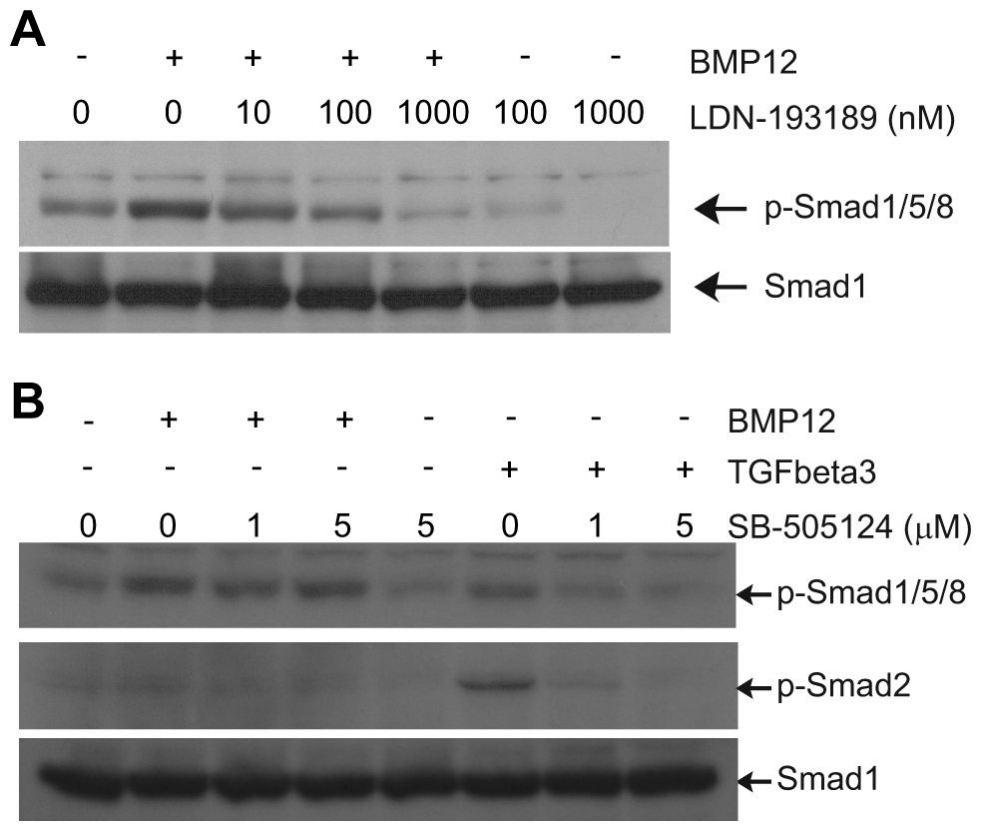
ALK2, 3, and 6 receptors are involved in BMP12 signaling. The BMP12-induced phosphorylation of Smad1/5/8 was dose-dependently blocked by the selective ALK2, 3, and 6 inhibitor LDN-193189 (A) but not by the ALK4, 5, and 7 inhibitor SB-505124 (B). The latter blocked the phosphorylation of Smad2 induced by TGFbeta3. Representative Western blots are shown. The Smad1 blots were used as sample loading controls.

The selective ALK2, 3, and 6 inhibitor LDN-193189 [41,42] or ALK4, 5, and 7 inhibitor SB-505124 [43] was then applied to ASCs to investigate their effects on BMP12-induced Smad1/5/8 phosphorylation. Western blots revealed that LDN-193189 dose-dependently inhibited both BMP12-induced as well as endogenous Smad1/5/8 phosphorylation (Figure 7A). SB-505124 blocked TGFbeta3-induced phosphorylation of Smad2 as well as phosphorylation of Smad1/5/8 in a dose-dependent manner; nevertheless, the drug did not affect BMP12-induced Smad1/5/8 phosphorylation (Figure 7B). Thus, ALK2, 3, and 6 rather than ALK4, 5, and 7 are likely to transmit BMP12 stimuli to the Smad1/5/8 signaling pathway.

## Discussion

To the best of our knowledge, this is the first in-depth study on the effect of BMP12 on ASC differentiation. Using tenomodulin, scleraxis, and the ScxGFP tendon reporter as tenogenic markers, we demonstrated that BMP12 is capable of dose- and time-dependently inducing tenogenic differentiation of ASCs. Results further revealed that BMP12 is likely to exert its effect via the ALK2, 3, and 6 type I receptors, and the latter in turn activate the Smad1/5/8 signaling pathway. In addition, a side-by-side comparison between BMP12 and BMP14 revealed that BMP12 was marginally better as a tenogenic factor. Together, our findings provide new insights into the cellular and molecular mechanisms of tenogenic differentiation as well as new basis for cell-based tendon repair.

The current study investigated the effects of BMP12 on ASC differentiation in comparison to BMP14. Our findings are largely in agreement with previous studies [44,45], reporting increased expression of scleraxis and tenomodulin at both protein and mRNA levels in BMP12-treated BM-MSCs [44] as well as BMP14-treated ASCs [45]. In the current study, BMP12 also induced the expression of scleraxis promoter-driven GFP in ASCs in a dose- and time-dependent manner, thus further demonstrating its role as a tenogenic cue. 

In addition to the tenogenic genes, we found that both BMP12 and BMP14 significantly augmented *ACAN* expression in ASCs, similar to observations reported by Park et al [45]. Nevertheless, the effect may not result in cartilage formation, as proteoglycans are also essential components of tendon [46], and indeed the resulting expression levels of ACAN in ASCs remained at least 10-fold lower than those detected in TFs. Furthermore, no increases in *COL2A1* were detected after either BMP12 or BMP14 treatment. In line with this, it has been shown that BMP14 is insufficient to directly induce chondrogenic differentiation and chondrocyte maturation in a micromass culture of limb bud mesenchyme cells [15], and BMP12 and BMP13 have been reported to have little impact on *COL2A1* expression in MC615 chondrocytes as well [13]. Future chondrogenic assays after longer term BMP12 treatment (e.g., 21 or 28 days) may be helpful to clarify the effect on BMP12 in chondrogenic differentiation of ASCs. 

In contrast to their effects on teno-chondrogenic marker gene expression, neither BMP12 nor BMP14 increased the osteogenic marker *OCN* and *RUNX-2* expression in ASCs. As a matter of fact, a dose- and time-dependant decrease in *OCN* expression was detected in the BMP12-treated ASCs. These findings further support the notion that both BMPs promote tenogenic rather than osteogenic differentiation of ASCs. Nevertheless, because osteogenesis was not the focus of this study, our analysis in this aspect is not definitive. Future evaluations are necessary to fully reveal the impact of BMP12 on osteogenic differentiation of ASCs. These studies may include examination of additional osteogenic marker such as osterix and alkaline phosphatase and mineralization assays. 

Our results demonstrated that BMP12 preferentially activates Smad1/5/8, but not Smad2/3 and p38 signaling. Interestingly, besides BMP12, the osteogenic factor BMP2 also activates Smad1/5/8. As discussed above, because our evaluation on osteogenesis in BMP12-treated ASCs is not definitive, we cannot completely rule out the involvement of BMP12 in osteogenic differentiation. However, our observation of a dose-dependent decrease of *OCN* in BMP12-treated ASCs is in sharp contrast to the substantial increase of *OCN* in stem cells exposed to BMP2 [13,14,47]. It is unclear why activation of the same pathway leads to the different cellular responses. One possible explanation is that BMP12 and BMP2 differentially activate individual Smad proteins within the Smad1/5/8 group and therefore result in different transcriptional responses. Smad1 [48,49] and Smad5 [49] signaling pathways have been associated with osteogenesis, whereas activated Smad8 was reported to directly induce tenogenic differentiation of MSCs *in vitro* and *in vivo* [50,51]. Thus, BMP12 may result in primarily Smad8 phosphorylation, while BMP2 may result in primarily Smad1 and Smad5 phosphorylation. Alternatively, the kinetics of Smad phosphorylation as well as the activities, stabilities, and localizations of phosphorylated Smads may be modified via activation of ligand-specific co-receptors or other non-canonical pathways [52,53], thus specifying the downstream transcriptional events. Additional studies are necessary to clarify the details of the signaling. 

This study further revealed that the BMP12-triggered phosphorylation of Smad1/5/8 was fully abolished by the ALK2, 3, and 6 blocker LDN-193189 but not by the ALK4, 5, and 7 inhibitor SB-505124. These findings, together with the overwhelming expression pattern of ALK6 mRNA in ASCs, lead to our hypothesis that BMP12 might preferentially signal through ALK6 to activate Smad1/5/8 pathway and therefore induce tenogenic differentiation of ASCs. In line with this hypothesis, it has been reported that BMP14 almost exclusively binds to the ALK6 type I receptor with high specificity both *in vivo* [54] and *in vitro* [55]. Comparing the sequences of human BMP14 (P43026) and BMP12 (NP_878248.2) revealed that the single Arg438 residue responsible for ALK6 binding specificity [55] is conserved between the two molecules. Moreover, overexpression of all seven ALK receptors demonstrated that ALK3 and ALK6 but not ALK1, 2, 4, 5, and 7 are capable of conveying BMP12 signaling [56]. Because LDN193189 at effective dosages is toxic to the cells in extended culture, we were unable to directly test its effect on BMP12-induced tenogenic differentiation of ASCs. Future studies using gene deletion or silencing will help to verify our signaling hypothesis and advance our understanding of the BMP12-induced tenogenic signaling pathway in ASCs.

 Our results demonstrate that ASCs have the capacity to differentiate into TF-like cells after BMP12 treatment; however, a concern remains that BMP12 may induce bone formation by ASCs *in vivo*. Although evidence provided from this study and from the literature [11–15] suggests that the risk for this is low, current data cannot exclude the possibility of osteogenesis. Zuk et al. [47] recently reported that ASCs may not respond to the osteogenic induction provided by BMP2, and speculated that this result was due to the lack of expression of the osteogenic response genes necessary for the BMP effect. In contrast, others groups have claimed that ASCs do indeed respond to BMP2 with osteogenic differentiation [57]. The disparate results may be due to the heterogeneous nature of ASCs or due to variations in cell culture conditions.

Taken together, this study demonstrated BMP12 as a potent tenogenic growth factor, inducing ASCs to form TF-like cells. Results further elucidated the signaling pathway leading to tenogenesis of ASCs. These findings provide a cellular and molecular basis for developing novel therapeutic strategies. For example, ASCs along with BMP12 can be delivered to the site of tendon injury during operative tendon repair to improve tendon healing or prevent tendon degeneration. 

## Supporting Information

Figure S1 **Canine ASCs (**A**) were induced to differentiate toward adipocytes (**B**), osteoblasts (**C**), and chondrocytes (**D**), showing positive staining for Oil Red O (**B**), Alizarin Red (**C**), and Alcian Blue (**D**), respectively.** Scale bar = 50 µm in A, B, D and 500 µm in C.
(TIF)

Figure S2 **The mRNA expression of *DCN* in ASCs was not affected by either BMP12 or BMP14 treatment.** The results were determined by quantitative real-time RT-PCR and are shown as fold change related to the expression level of control ASCs (dashed line).
(TIF)

Figure S3 **BMP12 had little effect on induction of Smad3 phosphorylation, while phosphorylated Smad3 (p-Smad3) was detected in TGFbeta3-treated ASCs.**
(TIF)

Figure S4 **All seven type I ALK receptors as well as the type II *BMPRII* genes were expressed in ASCs.** The results were detected by quantitative real-time RT-PCR and are shown as fold change related to the gene expression level in a reference total RNA sample (mRef) derived from 11 mouse cell lines.
(TIF)
