# Supplementary figures and images for: BMP12 induces tenogenic differentiation of adipose-derived stromal cells
Source: PLoS One. 2013 Oct 14;8(10):e77613. doi: 10.1371/journal.pone.0077613 (PMC3796462; doi:10.1371/journal.pone.0077613)

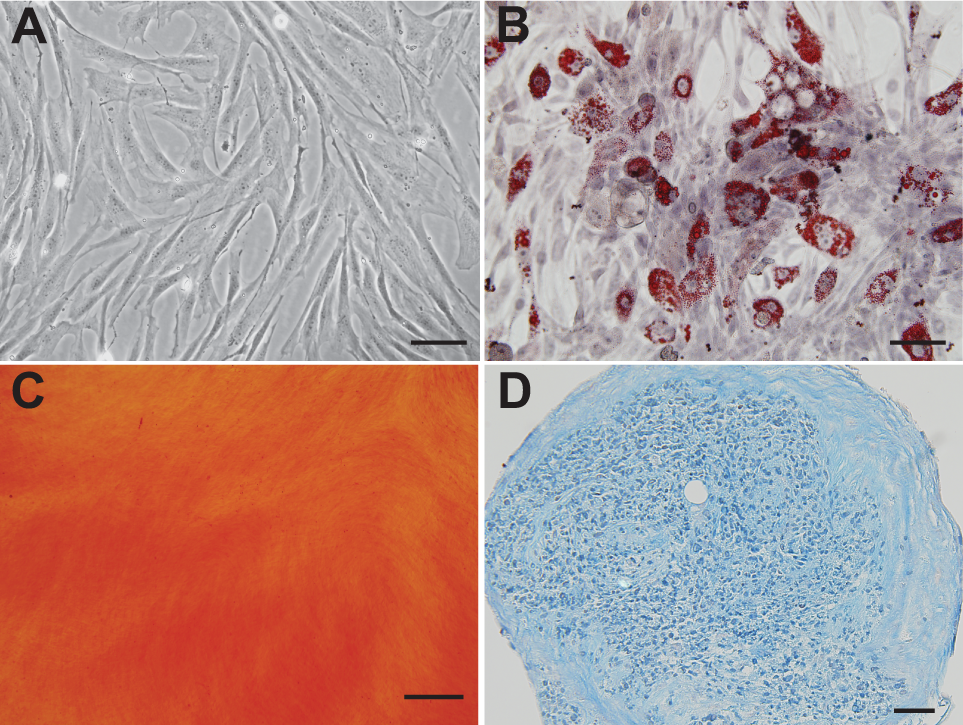

Supplement: Figure S1 — Canine ASCs (A) were induced to differentiate toward adipocytes (B), osteoblasts (C), and chondrocytes (D), showing positive staining for Oil Red O (B), Alizarin Red (C), and Alcian Blue (D), respectively. Scale bar = 50 µm in A, B, D and 500 µm in C. (TIF) [file pone.0077613.s001.tif]

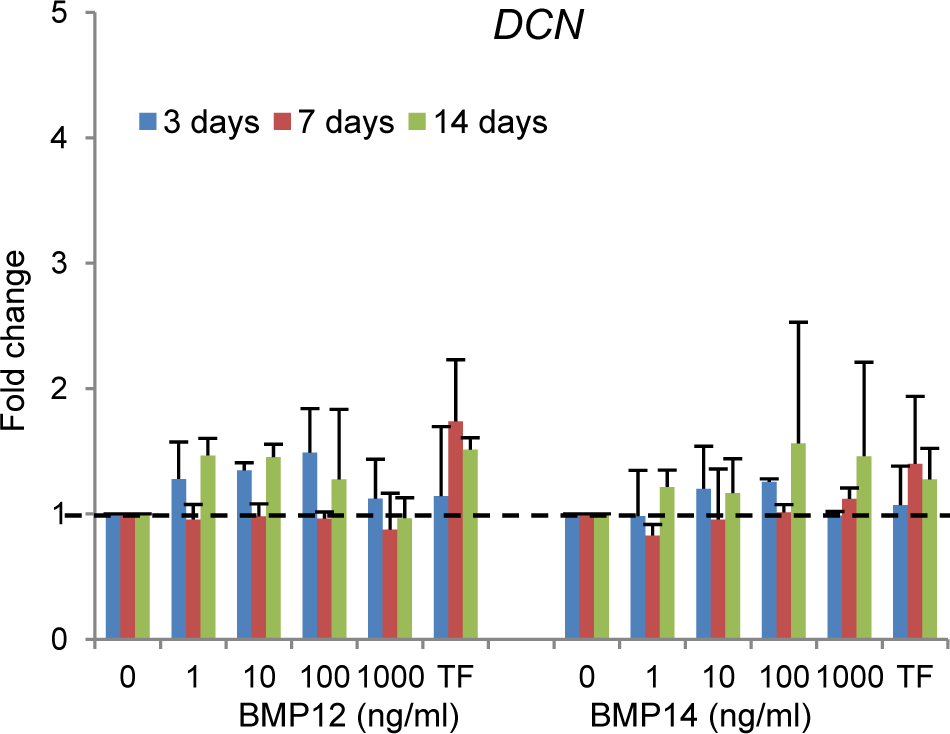

Supplement: Figure S2 — The mRNA expression of DCN in ASCs was not affected by either BMP12 or BMP14 treatment. The results were determined by quantitative real-time RT-PCR and are shown as fold change related to the expression level of control ASCs (dashed line). (TIF) [file pone.0077613.s002.tif]

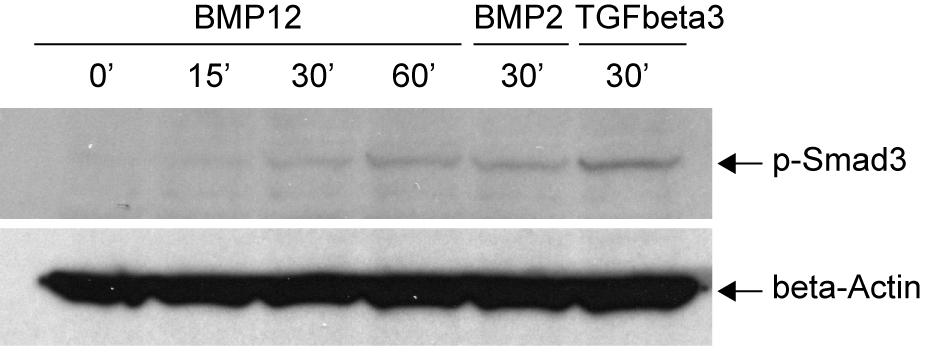

Supplement: Figure S3 — BMP12 had little effect on induction of Smad3 phosphorylation, while phosphorylated Smad3 (p-Smad3) was detected in TGFbeta3-treated ASCs. (TIF) [file pone.0077613.s003.tif]

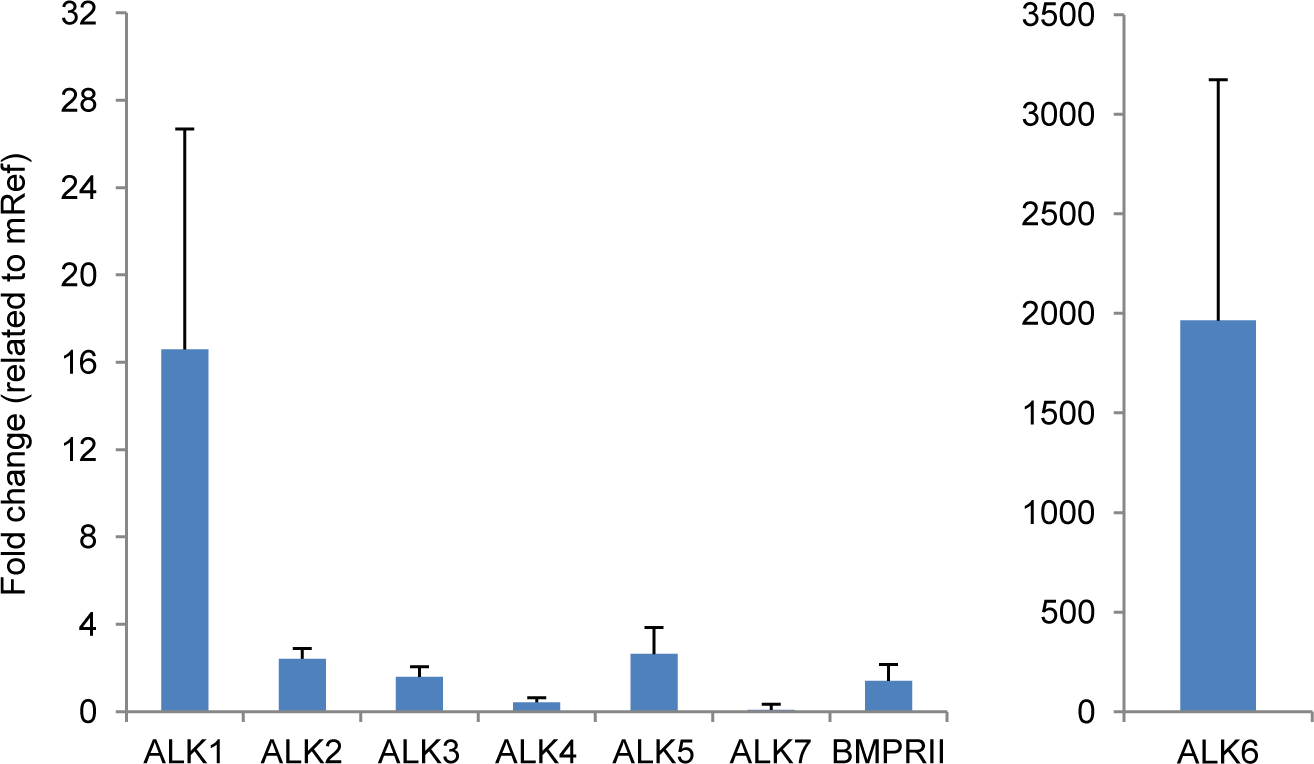

Supplement: Figure S4 — All seven type I ALK receptors as well as the type II BMPRII genes were expressed in ASCs. The results were detected by quantitative real-time RT-PCR and are shown as fold change related to the gene expression level in a reference total RNA sample (mRef) derived from 11 mouse cell lines. (TIF) [file pone.0077613.s004.tif]
